# Discrimination, Mental Health, and Readiness to Quit Smoking

**DOI:** 10.1177/10547738231183210

**Published:** 2023-06-26

**Authors:** Alexandria Jones-Patten, Sanghyuk S. Shin, Dawn T. Bounds, Adeline Nyamathi

**Affiliations:** 1 Columbia University Irving Medical Center, New York, NY, USA; 2 University of California Irvine, USA

**Keywords:** discrimination, cigarette smoking, homelessness, mental health, clinical research areas, African Americans

## Abstract

We conducted a cross-sectional study, examining the mediation effects of depression and anxiety on the association between discrimination and readiness to quit cigarette smoking among African American adult cigarette smokers experiencing homelessness. Using a convenience sample, participants were recruited from a homeless shelter in Southern California. Scores of discrimination, depressive, and anxiety symptoms, and readiness to quit smoking were analyzed using linear regression modeling. We enrolled 100 participants; 58 participants were male. In the final model, discrimination had no association with readiness to quit (*b* = 0.02; 95% CI [−0.04, 0.08]; *p* = 0.47). The indirect effects of depression (*b* = 0.04, [0.01, 0.07]; *p* = 0.02) and anxiety (*b* = 0.03; [0.01, 0.05]; *p* = 0.04) reached statistical significance; the direct effects of depression (*b* = −0.01; [−0.09, 0.04]; *p* = 0.70) and anxiety (*b* = −0.00; [−0.09, 0.06]; *p* = 0.86) did not. Future studies should explore these associations to enhance smoking cessation programs for this population.

## Background

### Cigarette Smoking Among Persons Experiencing Homelessness

An estimated 568,000 people experienced homelessness (PEH) in the United States in 2019. African Americans (AAs) disproportionately represent this population, as 40% of PEH in the United States identified as AA (Henry et al., 2020). Factors associated with homelessness often include poverty, unemployment, discrimination experiences, incarceration, and high housing costs. Roughly 73% to 80% of PEH reportedly smoke cigarettes (Adhikari et al., 2015; Baggett & Rigotti, 2010; Tsai & Rosenheck, 2012). Adverse health outcomes are also exacerbated among PEH. For example, cigarette smoking has deleterious effects on the cardiovascular system, including altering the structure, function, and activation of platelets, thereby increasing the risk of thrombosis, and increasing the risk of atrial and ventricular arrhythmias (Morris et al., 2015). The high prevalence of smoking among PEH may be largely responsible for increased risk of cardiovascular disease, which is threefold higher in PEH compared to housed individuals (Al-Shakarchi et al., 2020). (Baggett et al., 2018).

Among current PEH smokers, one-third report feelings of readiness to quit cigarette smoking within the next 6 months; almost one-fifth report readiness to quit within the next month (Garey et al., 2015). The motivation to initiate quitting cigarette smoking could be strained by several variables. Among the general population, current cigarette smoking, including nicotine dependence, has been positively associated with everyday discrimination (Kendzor et al., 2014; Sims et al., 2016), while depressive symptoms have been linked to a decline in the likelihood of achieving smoking cessation (Catley et al., 2005). As a behavior among PEH, cigarette smoking continuance is linked to high levels of boredom and stress (Okuyemi et al., 2006), and anxiety (Chen et al., 2016), suggesting cigarette smoking may serve as a protective measure for coping with chronic stressors such as discrimination. Barriers to smoking cessation specifically in AA PEH may include low self-efficacy (Pinsker et al., 2018), nicotine dependence, homelessness, and fatalism (Vijayaraghavan et al., 2018). Several studies have measured quit attempts among PEH (Akande et al., 2020; Businelle et al., 2014; Savoy et al., 2021), but there is a dearth of knowledge regarding the influence of discrimination and mental health on readiness to quit cigarette smoking among PEH, specifically among AA PEH. The aims of this paper are (a) to explore the association between discrimination and readiness to quit cigarette smoking among AA PEH and (b) analyze whether symptoms of depression or anxiety mediate this association.

### Discrimination, Depression, and Anxiety in PEH

Discrimination experiences among PEH are endemic and may contribute to the causes of homelessness (Otiniano Verissimo et al., 2021). In one study of PEH, AA participants were more likely than Whites to report at least one perceived discrimination experience in their lifetime (79.1% AA vs. 67.7% White; *p* = .007); moreover, AA PEH experience increased burden on life because of perceived discrimination (Wrighting et al., 2019). Another study identified reasons for perceived discrimination in older AA PEH which included both experiencing racism early in life and structural racism, including discrimination in the criminal justice system, exposure to violence and criminal activity, and limited family wealth and employment opportunities (Paul et al., 2020). These exposures to discrimination may impact mental health outcomes (Clark et al., 2015) and accelerate engagement in the maladaptive coping mechanism of cigarette smoking (Caceres et al., 2021; Parker et al., 2017).

Depression affects nearly half (46.7%) of PEH (Ayano et al., 2021). Depressive symptoms among various samples of PEH in smoking cessation programs have been explored previously. In one randomized control trial of PEH (*n* = 431), the association between depression and psychological distress was assessed during enrollment in a smoking cessation program. The investigators found, at week 26, depression was associated with increased craving to smoke cigarettes (*r* = .26, *p* < .001), lower perceived stress (*r* = .14, *p* = .013), hopelessness (*r* = −.33, *p* < .001), and less confidence in quitting (*r* = .26, *p* = .002) (Robinson et al., 2016). These data suggest that depressive symptoms and increased cravings could lead to increased heaviness of smoking, which may impact readiness to quit cigarette smoking. Depressive symptoms could also be influenced by discrimination experiences (Scheuermann et al., 2020), which may further impact smoking abstinence.

Anxiety may also be associated with cigarette smoking, but literature analyzing this association is limited. One study measuring past month pain severity and heaviness of smoking among homeless adults (*n* = 461) found that anxiety moderated this association (*b* = .005; *p* = .040) (Reuven et al., 2021). Elsewhere, a study reviewing the perceived barriers to smoking cessation among 100 adult PEH found that the use of cigarettes to relieve anxiety was the most common reason for continued smoking, followed by cravings and lack of resources for smoking cessation programs/support (Chen, et al., 2016). Furthermore, research suggests discrimination experiences may also impact anxiety among AAs (Mouzon et al., 2017), but this association among AA PEH who smoke cigarettes should be investigated further.

### Readiness to Quit Cigarette Smoking and Substance Use Among PEH

Quitting smoking while experiencing homelessness may present other challenges specific to the experience of homelessness itself. In a study of 40 PEH (80% AA sample) using nicotine replacement therapy to assist smoking cessation, participants described feeling pressure from their surroundings to smoke and drink in and around shelters, leading some to start or resume smoking (Pratt et al., 2019). In another study of 31 equally proportioned White and AA PEH residing in family shelters, significant barriers to quitting smoking included the ubiquity of cigarette smoking and its central role in social interactions among residents in the family shelter setting, and its importance as a coping mechanism (Stewart et al., 2015). The data suggest there is a competing interest between quitting cigarette smoking and maintaining the social support of others in the immediate environment.

Even among those who may want to quit cigarette smoking, other substances may be used in place of cigarettes. In a sample of cigarette smokers experiencing homelessness (*n* = 396), two-thirds (67.2%) reported concurrent use of cigarettes with other substances (Neisler et al., 2018a). Furthermore, studies of smoking cessation programs among PEH suggest higher rates of successful smoking cessation in Whites compared to AAs (AA: 14.3% vs. White: 24.4%; *p* = .007; Nollen et al. [2019] and AA vs. White; odds ratio [OR]: 0.84; *p* = .002; Stevens et al. [2016]). Success in smoking cessation among AAs may be influenced by several psychosocial factors, including racism and discrimination. Experiences of everyday discrimination have been associated with alcohol and illicit drug use disorders (Clark et al., 2015) and current cigarette smoking (Forde et al., 2021) among the general AA population.

### Summary of Gaps in Current Knowledge

AAs are largely overrepresented in the U.S. homeless population, and cigarette smoking among PEH is highly prevalent. Smoking cessation programs have been utilized in populations of PEH, but AAs tend to be less successful in quitting smoking than their White counterparts. The existence of an association between experiences of discrimination and readiness to quit cigarette smoking in AA PEH is unclear. For example, perceived discrimination in AAs has been explored (Bello et al., 2021; Paul et al., 2020; Purnell et al., 2012; Wrighting et al., 2019), but these studies do not measure readiness to quit cigarette smoking. While depression and anxiety have each been associated with smoking habits in PEH; to our knowledge, no studies have explored whether depression or anxiety mediate this association. Our study aims to (a) investigate whether discrimination is associated with readiness to quit cigarette smoking among AA PEH and (b) conduct a mediation analysis to determine whether depressive or anxiety symptoms mediate this association. We hypothesize that discrimination experiences will have an inverse relationship with readiness to quit cigarette smoking, and that depressive and anxiety symptoms will independently mediate the association between discrimination experiences and readiness to quit cigarette smoking.

### Conceptual Framework

Our conceptual framework is driven by DiClemente and Prochaska’s (1991) transtheoretical model (TTM), also known as the stages of change model. DiClemente and Prochaska suggest individuals move through six stages of change when attempting to make a behavior change, such as quitting cigarette smoking. The first three stages include the following: Precontemplation—there is no intention to quit smoking in the next 6 months; Contemplation—there are plans to quit smoking in the next 6 months; and Preparation—the person plans to quit smoking in the next 30 days. Between these stages, there is a shift from inaction to preparing to take action. In the Action stage, a quit attempt occurs; while the fifth stage is Maintenance, wherein the quit attempt is becoming less of a struggle and more of a routine. Individuals may or may not enter the Termination (sixth) stage, in which a relapse occurs. The TTM has been used among studies exploring substance use and readiness to quit among homeless adults (Adhikari et al., 2015; Velasquez et al., 2000).

## Methods

### Study Design, Setting, and Participants

The goal of this study is to investigate whether discrimination is associated with readiness to quit cigarette smoking and the role of depressive or anxiety symptoms in mediating this association. A detailed description of the design, setting, and participants has been discussed elsewhere (Jones-Patten et al., 2023). Briefly, this study used a cross-sectional survey design to explore the association between perceived discrimination and readiness to quit cigarette smoking. In addition, depression and anxiety were independently measured as mediators on the association between discrimination and readiness to quit cigarette smoking. A sociodemographic questionnaire was also used to collect descriptive statistics of participants’ age, gender, and homeless and smoking histories. Participants from a large homeless shelter in Southern California were surveyed.

### Inclusion and Exclusion Criteria

Participants were included in the study if they were at least 18 years of age, identified as AA, self-reported being a current cigarette smoker, were experiencing homelessness at least 1 month, and provided verbal consent. Study participants were asked if they have smoked at least 100 cigarettes in their lifetime and “how many cigarettes have you smoked in the last 7 days?” Participants who reported smoking at least 100 cigarettes in their lifetime and at least one cigarette within the last 7 days were included in the study. Potential participants were excluded if they were cognitively impaired, as assessed by a decision-making capacity tool during the screening process, to ensure the participant understood the research study and their rights as a participant.

### Data Collection

Following institutional review board approval, data for the current study were collected from February to June, 2022. Participants were screened in a private room. Interested participants were given a study information sheet. Next, a screener questionnaire was completed by the lead author, who asked inclusionary criteria questions. Participants received $3 for participation in the screener questionnaire and participant contact information and verbal consent to participate were collected. An iPad was used to input survey responses. Research Electronic Data Capture was used to collect and store this data. Following completion of the survey, participants received an additional $15.

### Measures

#### Dependent Variable

##### Readiness to Quit

The contemplation ladder is a 10-step ladder, with every other step representing a smoker’s thoughts on quitting smoking. The ladder ranges from 0 to 10, with 0 classified as “no thought of quitting,” and 10 meaning “taking action to quit” (Biener & Abrams, 1991). The validity of this scale has been assessed among a sample of smokers (Amodei & Lamb, 2004).

#### Independent Variable

##### Perceived Discrimination

The Everyday Discrimination Scale, (Williams et al., 1997) is a nine-item instrument measuring day-to-day experiences of discrimination using a six-point Likert scale. Responses range from “1” (never) to “6” (almost every day), and total scores range from 9 to 60, with higher scores indicating greater perceived discrimination. A follow-up question was asked only of those answering “A few times a year” or more frequently to at least one question: What do you think is the main reason for these experiences? If participants mentioned more than one option, all options stated by the participant were checked. “Homelessness” as an additional option was added to this unweighted follow-up question by this study’s authors. This scale has demonstrated good internal consistency elsewhere (Williams et al., 1997). In our study, the Cronbach’s alpha was .86.

#### Mediators

##### Depression

The Centers for Epidemiologic Studies-Depression Scale is a 20-item survey, using a four-point system to measure depressive symptoms over the prior 7 days (Radloff, 1977). Scores range from 0 to 60, with scores greater than 15 indicating depressive symptomatology. This scale has been assessed for reliability and validity in PEH, previously (Fitzpatrick et al., 2015; Nyamathi et al, 2012; Wong, 2000). Participants with scores of 16 or greater were referred to mental health services. The Cronbach’s alpha for our study was .88.

#### Anxiety

The Generalized Anxiety Disorder Scale-7 is a seven-item, self-rated scale, using a four-point Likert scale, to assess the severity of anxiety symptoms (Spitzer et al., 2006). Scores range from 0 to 21; scores of 10 or greater indicate a moderate level of anxiety. Participants with anxiety scores of 10 or greater were also referred to mental health services. For our study, the Cronbach’s alpha was .89. Rutter and Brown (2017) have previously reported on the scale’s reliability in a sample of adult men and women (Cronbach’s alpha: .85) (Rutter, 2017).

#### Potential Confounding Variables

##### Texas Christian University Drug Screen V

The 17-item Texas Christian University (TCU) Drug Screen V identifies substance use, including the frequency of use (Knight et al., 2018). Scores range from 0 to 11. Items 12 through 17 are not included as part of the total score. For item 13, “How often did you use each type of drug in the last 12 months,” we combined the original five response options (“Daily,” “1–5 times per week,” “1–3 times per month,” “Only a few times,” and “Never”) to three (“Daily Use,” “Some Use,” and “No Use”) (Table 1). The TCU Drug Screen V scoring is as follows: Mild disorder: 2 to 3 points, Moderate disorder: 4 to 5 points, and Severe disorder: 6 or more points. Prior research demonstrates the reliability of this scale among adults (Knight et al., 2018). The Cronbach’s alpha for this study was .88.

**Table 1.** Demographics of AA Individuals Experiencing Homelessness.

Sample Characteristics	Male (*N* = 58)	Female (*N* = 42)	Overall (*N* = 100)
Age			
Mean (SD)	49.5 (14.3)	49.7 (12.3)	49.6 (13.4)
Median [Min, Max]	53.0 [23.0, 80.0]	54.0 [20.0, 68.0]	53.5 [20.0, 80.0]
Education			
Less than high school	20 (34.5%)	13 (31.0%)	33 (33.0%)
High school completion	17 (29.3%)	14 (33.3%)	31 (31.0%)
Trade school, some college or college completion	18 (31.0%)	7 (16.7%)	25 (25.0%)
Missing	3 (5.2%)	8 (19.0%)	11 (11.0%)
Employment			
Employed	9 (15.5%)	3 (7.1%)	12 (12.0%)
Retired	8 (13.8%)	2 (4.8%)	10 (10.0%)
Seeking opportunities	21 (36.2%)	14 (33.3%)	35 (35.0%)
Unemployed	15 (25.9%)	15 (35.7%)	30 (30.0%)
Missing	5 (8.6%)	8 (19.0%)	13 (13.0%)
Everyday discrimination scores			
Mean (SD)	32.7 (10.8)	32.7 (12.4)	32.7 (11.4)
Median [Min, Max]	35.0 [10.0, 54.0]	35.5 [9.00, 54.0]	35.0 [9.00, 54.0]
Readiness to quit scores			
Mean (SD)	6.02 (2.93)	5.43 (3.56)	5.77 (3.21)
Median [Min, Max]	5.00 [0, 10.0]	6.00 [0, 10.0]	5.50 [0, 10.0]
Time to first cigarette			
(a) Within 5 min (3 points)	19 (32.8%)	20 (47.6%)	39 (39.0%)
(b) Within 6–30 min (2 points)	16 (27.6%)	14 (33.3%)	30 (30.0%)
(c) Within 31–60 min (1 point)	8 (13.8%)	3 (7.1%)	11 (11.0%)
(d) After 60 min (0 points)	15 (25.9%)	5 (11.9%)	20 (20.0%)
Cigarettes smoked per day			
(a) 10 or less (0 points)	40 (69.0%)	23 (54.8%)	63 (63.0%)
(b) 11–20 (1 point)	14 (24.1%)	15 (35.7%)	29 (29.0%)
(c) 21–30 (2 points)	1 (1.7%)	2 (4.8%)	3 (3.0%)
(d) 31 or more (3 points)	3 (5.2%)	2 (4.8%)	5 (5.0%)
Depression scores			
<16	16 (27.6%)	8 (19.0%)	24 (24.0%)
≥16	42 (72.4%)	34 (81.0%)	76 (76.0%)
Anxiety scores			
<10	18 (31.0%)	17 (40.5%)	35 (35.0%)
≥10	40 (69.0%)	25 (59.5%)	65 (65.0%)
TCU drug scores			
Mean (SD)	3.09 (3.27)	3.64 (3.53)	3.32 (3.38)
Median [Min, Max]	3.00 [0, 10.0]	3.00 [0, 11.0]	3.00 [0, 11.0]
Alcohol use			
No use in last 12 months	33 (56.9%)	25 (59.5%)	58 (58.0%)
Some use in last 12 months	17 (29.3%)	9 (21.4%)	26 (26.0%)
Daily use in last 12 months	8 (13.8%)	8 (19.0%)	16 (16.0%)
Marijuana use			
No use in last 12 months	33 (56.9%)	30 (71.4%)	63 (63.0%)
Some use in last 12 months	10 (17.2%)	6 (14.3%)	16 (16.0%)
Daily use in last 12 months	15 (25.9%)	6 (14.3%)	21 (21.0%)
Crack cocaine use			
No use in last 12 months	53 (91.4%)	33 (78.6%)	86 (86.0%)
Some use in last 12 months	3 (5.2%)	2 (4.8%)	5 (5.0%)
Daily use in last 12 months	2 (3.4%)	7 (16.7%)	9 (9.0%)
Methamphetamine use			
No use in last 12 months	51 (87.9%)	39 (92.9%)	90 (90.0%)
Some use in last 12 months	5 (8.6%)	1 (2.4%)	6 (6.0%)
Daily use in last 12 months	2 (3.4%)	2 (4.8%)	4 (4.0%)

*Note*. AA = African Americans; TCU = Texas Christian University.

##### Smoking Intensity

The Heaviness of Smoking Index (Heatherton et al., 1989) is a measure used to express cumulative smoking exposure. It consists of two items from the Fagerstrom Test for Nicotine Dependence: (a) How soon after you wake up do you smoke your first cigarette? and (b) How many cigarettes per day do you smoke? The index is calculated using the sum of the scores on those two items. Response options for time to the first cigarette of the day are as follows: within 5 min (3 points), within 6 to 30 min (2 points), 31 to 60 min (1 point), and after 60 min (0 points). Response options for average daily number of cigarettes smoked are as follows: 10 or less (0 points), 11 to 20 (1 point), 21 to 30 (2 points), and 31 or more (3 points). Participants will fall into one of three categories for nicotine dependence: low (0–1), medium (2–4), and high (5–6). The results of the individual questions are reflected in Table 1. The HSI has demonstrated its reliability to predict quitting smoking behaviors in prior research (Borland et al., 2010).

### Demographics

Sample characteristics that were collected include age, gender, education, and employment. Age was measured in years. Gender was recorded as either male or female. Participants’ education was categorized as follows: “Less than High School,” “High School Completion,” “Trade School experience,” “some college,” and “college completion.” Similarly, employment categories included “Employed,” “Seeking opportunities,” “Retired,” “Unemployed,” or “Prefer not to say.”

### Data Analysis

Both the primary independent variable and the primary outcome variable were analyzed as continuous variables. Although we enrolled 100 participants to ensure sufficient power for fitting multilinear regression modeling, we estimated an enrollment sample of 84, using a power of 80% and two-sided alpha at 5% to detect statistical significance between everyday discrimination and contemplation ladder scores, assuming *r* = .3. We performed descriptive statistics for dependent and independent variables. Subsequently, linear regression modeling was used to identify associations between everyday discrimination and readiness to quit cigarette smoking.

Potential confounders in this model were age, education, heaviness of smoking, and TCU Drug Screen V scores. We used a change-in-estimate approach to select the variables included in the final model (Greenland, 1989), with our final model including only the variables that led to a change in the beta estimate for everyday discrimination scores by 10% or greater. Subsequently, we conducted a mediation analysis and adjusted for confounding in the mediation model (Tingley et al., 2014).

In the mediation analysis, depression and anxiety were each conducted in separate models to determine if either mediated the association between everyday discrimination and readiness to quit. We used the bootstrapping method for causal mediation analysis. First, we ran the unadjusted bivariate model for everyday discrimination and the selected mediator (depression or anxiety). Then we ran the same model and added the dependent variable. Next, we used the mediation function on the first two models to determine the total, direct and indirect effects, while controlling for confounding variables. Data were computed using R version 4.2.0 (R Core Team, 2020).

## Results

### Sociodemographic Characteristics

Sample characteristics are described in Table 1. Of the 100 participants enrolled, over half (*n* = 58) of participants were male, and the mean age was 49.6 years (SD = 13.4). One-third of participants (33.0%) had less than a high school education, and more than one-third of participants (35.0%) were seeking employment opportunities.

#### Perceived Discrimination

The mean everyday discrimination scores for both men (32.7, SD = 10.8) and women (32.7, SD = 12.4) were similar. When responding to the question regarding the main reason for discrimination experiences, 43% of the sample reported “race” as the main reason for experiencing discrimination, while 37% of participants reported “homelessness.” Additional reasons participants selected for discrimination included gender (16%), shade of skin color (16%), age (15%), and religion (10%).

#### Depressive and Anxiety Symptoms

Overall, 72.4% (*n* = 42) of men and 81.0% (*n* = 34) of women had depression scores of 16 or higher. Discrimination scores were higher among those with depressive symptoms of 16 or greater (mean: 35.03) compared to those with scores under 16 (mean: 25.33; *p* = .001). For anxiety symptoms, 69.0% (*n* = 40) of men and 59.5% (*n* = 25) of women had anxiety scores of 10 or more. Discrimination scores were higher among those with anxiety scale scores of 10 or more (mean: 35.0) compared to those with scores less than 10 (mean: 28.5; *p* = .01).

#### Cigarette Use and Readiness to Quit

Contemplation ladder scores were not significantly different among men (6.02, SD = 2.93) and women (5.43, SD = 3.56). More than one-third (39%) of participants reported smoking their first cigarette within 5 min of waking; another 30% reported smoking their first cigarette within 6 to 30 min of waking. The majority of participants reported smoking either 10 cigarettes or less (63%) or 11 to 20 cigarettes (29%) each day.

#### Other Substance Use

There were no significant differences in gender between TCU scores (men: 3.09 vs. women: 3.64). A combined 42% of all participants reported some or daily alcohol use within the last 12 months, while 37% reported either some or daily marijuana use in the last 12 months; 14% reported some or daily use of crack cocaine, and 10% reported some or daily use of methamphetamine.

#### Discrimination and Readiness to Quit Cigarette Smoking

Table 2 displays the unadjusted and adjusted analyses of everyday discrimination and contemplation ladder scores. In the unadjusted analysis, there was no association between everyday discrimination scores and contemplation ladder scores (*b* = 0.01; 95% CI [−0.04, 0.01]; *p* = .70). Compared to those with less education, participants with trade school or college education had higher contemplation ladder scores (*b* = 2.10; [0.48, 3.80]; *p* = .01). Using the change in estimate approach, education and Heaviness of Smoking Index scores each changed the beta estimate of everyday discrimination by more than 10%. In the final model, after adjusting for education and heaviness of smoking, everyday discrimination scores had no association with contemplation ladder scores (*b* = 0.02; [−0.04, 0.08]; *p* = .47). In addition, education remained significantly associated with higher contemplation ladder scores (*b* = 1.99; [0.26, 3.72]; *p* = .02).

**Table 2.** Bivariate and Multivariate Linear Regression Model Results of Everyday Discrimination and Outcome Variable Contemplation Ladder Scores.

Variables	Unadjusted beta	[95% CI]	*p* Value	* Adjusted beta	[95% CI]	*p* Value
Age	−.01	[−0.06, 0.03]	.6	Not Included	—	—
Gender (male)	Ref.	—	—	Not Included	—	—
Female	−.59	[−1.90, 0.70]	.4	Not Included	—	—
Education (less than high school)	Ref.	—	—	Ref.	—	—
High school	.80	[−0.77, 2.4]	.3	.84	[−0.81, 2.50]	.31
Trade school, some college or college completion	2.10	[0.48, 3.80]	** (.01)	1.99	[0.26, 3.72]	**.02
Everyday discrimination scores	.01	[−0.04, 0.01]	.70	.02	[−0.04, 0.08]	.47
TCU scores	.03	[−0.16, 0.22]	.70	Not Included	—	—
Heaviness of Smoking Index	−.30	[−0.70, 0.10]	(.14)	−.21	[−0.64, 0.21]	.32

*Note*. TCU = Texas Christian University.

* Adjusted for education and heaviness of smoking.

** *p* Value of <.05 indicates statistical significance.

#### Mediation Analysis

A mediation analysis was conducted for depression and anxiety separately. We found the association between everyday discrimination scores and depression scores to be statistically significant (*b* = 0.54, *p* < .001). When modeling depression as a mediator (Table 3) and adjusting for education and heaviness of smoking scores, the indirect effect was significant (*b* = 0.04, 95% CI [0.01, 0.07]; *p* = .02). The total effect (*b* = 0.02; [−0.05, 0.08]; *p* = .56) and direct effect (*b* = −0.01; [−0.09, 0.04]; *p* = .70) did not reach statistical significance.

**Table 3.** Mediation Analysis (Depression) Results of Everyday Discrimination and Outcome Variable Contemplation Ladder Scores.

Mediator (Depression)	Unadjusted beta	[95% CI]	*p* Value	*Adjusted beta	[95% CI]	*p* Value
Total effect	*b* = .01	[−0.04, 0.05]	.54	.02	[−0.05, 0.08]	.56
Direct effect	*b* = −.01	[−0.06, 0.04]	.82	−.01	[−0.09, 0.04]	.70
Indirect effect	*b* = .03	[−0.00, 0.05]	.10	.04	[0.01, 0.07]	**.02

* Adjusted for education and heaviness of smoking.

** *p* Value of <.05 indicates statistical significance.

The association between everyday discrimination scores and anxiety scores was statistically significant (*b* = 0.23, *p* < .001). In the mediation analysis including anxiety as a mediator (Table 4), adjusting for education and Heaviness of Smoking Index, the indirect effect between discrimination scores and contemplation ladder scores was statistically significant (*b* = 0.03; 95% CI [0.01, 0.05]; *p* = .04). The total effect (*b* = 0.02; [−0.04, 0.08]; *p* = .56) and direct effect (*b* = −0.00; [−0.09, 0.06]; *p* = .86) did not reach statistical significance.

**Table 4.** Mediation Analysis (Anxiety) Results of Everyday Discrimination and Outcome Variable Contemplation Ladder Scores.

Mediator (Anxiety)	Unadjusted beta	[95% CI]	*p* Value	*Adjusted beta	[95% CI]	*p* Value
Total effect	.01	[−0.04, 0.05]	.54	.02	[−0.04, 0.08]	.56
Direct effect	−.01	[−0.07, 0.03]	.86	−.00	[−0.09, 0.06]	.86
Indirect effect	.02	[−0.00, 0.05]	.06	.03	[0.01, 0.05]	**.04

* Adjusted for education and heaviness of smoking.

** *p* Value of <.05 indicates statistical significance.

## Discussion

To build on the knowledge of research exploring cigarette smoking among homeless populations, we investigated whether discrimination experiences are associated with readiness to quit cigarette smoking and whether mental health outcomes depression and anxiety mediated this association. Our study did not find an association between discrimination experiences and readiness to quit cigarette smoking in the main effects model that does not consider mediation. When accounting for the indirect effect through depression and anxiety separately in mediation analyses, we found an association between discrimination and readiness to quit cigarette smoking, even though the total effects did not reach statistical significance. Moreover, contrary to our hypothesis, the mediated association between discrimination and readiness to quit cigarette smoking was positive.

To our knowledge, this is the first study to measure these associations among a sample of AA PEH. Our results did demonstrate that both women and women were experiencing a moderate amount of discrimination. These experiences could stem from their housing status or identifying as a racial minority, but future studies would need to confirm this. While discrimination has been linked to many negative outcomes, its effect on permanently abstaining from cigarette smoking may be more complex, including readiness to quit smoking among some PEH smokers. However, interventions specifically tailored to AA PEH to manage and appropriately respond to discrimination experiences may be warranted.

Discrimination was positively associated with depressive and anxiety symptoms, independently in our mediation analysis. Other studies have demonstrated an association between discrimination experiences and depressive/anxiety symptoms among AA previously (Goodwill et al., 2021; Mouzon et al., 2017), but they were not among a sample of AA PEH who smoke cigarettes. Our study builds on this gap in literature, as we address this particular association in more detail elsewhere (Jones-Patten et al., 2023).

The experience of different forms of discrimination coupled with the stress of surviving on the streets could lead to higher levels of depression and anxiety over time. Depressive symptoms among PEH have been associated with younger age (<25 years), while major depressive disorder is higher among PEH 50 and older (Ayano et al., 2021). Chen et al. (2016) found that PEH cigarette smokers admitted to using cigarettes to manage chronic stress and anxiety. Among our sample, the average age was slightly below 50. Higher rates of depressive and anxiety disorders may be the result of both chronic exposure to discrimination and chronic episodes of experiencing homelessness and should be addressed in future studies examining discrimination and depressive and anxiety disorders over time among PEH.

The traditional method for conducting a mediation analysis is to consider mediation effects only when an effect of the independent variable on the dependent variable exists (Baron & Kenny, 1986). This has been challenged by several authors (O’Rourke & MacKinnon, 2018; Zhao et al., 2010), who argue that if either the indirect and direct paths are positive and the other negative, then testing the association between the independent and dependent variables may fail to find statistical significance. In our case, we found that a mediated effect exists, but without a statistically significant direct effect, also known as indirect-only mediation (Zhao et al., 2010). In this instance, the direct effect between everyday discrimination and readiness to quit was not statistically significant but had a negative association. This negative association may have masked the positive indirect effects through depression and anxiety when estimating the total effect.

In addition, our study showed a positive association with depressive/anxiety symptoms and readiness to quit cigarette smoking. This is inconsistent with previous literature. Ojo-Fati et al. (2016) found that among a sample of persons experiencing homelessness enrolled in a randomized control trial for smoking cessation (Power to Quit), smokers who were depressed at baseline had lower confidence to quit (OR = 1.10, 95% CI [1.01, 1.19], *p* = .04), and were less motivated to adhere to nicotine replacement therapy (OR = 1.04, [1.00, 1.07], *p* = .04). One explanation may be that an individual’s environment influence both depressive and anxiety symptoms as well as readiness to quit. Pratt et al. (2019) found that smokers (*n* = 40) in a smoking cessation RCT described both pressures in and around shelters to smoke and the stress associated with experiencing homelessness challenged their attempt(s) to quit cigarette smoking. Given that we did not measure social or environmental support, we cannot determine to what extent the environment of our sample participants impacted the association between depressive/anxiety symptoms and readiness to quit cigarette smoking. Future research should examine these variables longitudinally to more effectively determine an association.

Significant confounders in our study included education and heaviness of smoking. Lower levels of education have reportedly been associated with current smoking (Parker et al., 2016; Sartor et al., 2021). Among AAs, the education-level disparity between ever-smokers (those who have smoked at least 100 cigarettes in their lifetime) with less than a high school education and those who graduated college increased from a 2.2-fold difference in 1992–1993 to a fourfold difference in 2018 (Nguyen-Grozavu et al., 2020). It may be beneficial to introduce education about the deleterious effects of cigarette smoking to younger age groups. In addition, heaviness of smoking specifically among homeless smokers has been negatively associated with successful quit attempts lasting more than 24 hours (Neisler et al., 2018b) and number of quit attempts (*r* = .29, *p* < .05) (Akande et al., 2020). Our study lends to future research in this area, suggesting that barriers to successfully abstaining from cigarette smoking among AA PEH are multifactorial, in which resources for smoking cessation will need to be tailored to the individual, and cigarette smoking may be a protective measure for this population.

## Limitations

While this study adds to the literature surrounding smoking cessation among a vulnerable population, it comes with limitations. First, our sample is a convenience sample rather than a random sample. In addition, our sample is all AA, which may not explain associations between discrimination and readiness to quit cigarette smoking among other minority groups experiencing homelessness. It should not be discounted, however, that AAs largely overrepresent PEH in the United States. Second, we used cross-sectional data, so caution is warranted when inferring a causal relationship. Third, we did not measure quit attempts, which may have better-explained readiness to quit. Fourth, we collected self-report measures for everyday discrimination, depressive and anxiety symptoms, smoking status, housing status, and race. While this study was conducted in a shelter requiring ID badges to be worn at all times for staff and residents, we did not confirm badges with shelter personnel prior to enrolling participants. Participants were, however, recruited primarily from designated resident smoking areas within the shelter.

## Conclusion

This study discusses discrimination experiences and psychosocial factors impacting readiness to quit cigarette smoking among homeless AA adults. While smoking cessation programs have been offered for this population, it is important to note that cigarette smoking may be a way to cope with many circumstances not typically supported in smoking cessation programs, including symptoms of anxiety or depression, isolation, and the overall experience of homelessness. Smoking cessation programs should analyze these barriers and provide resources that enhance resilience among participants, further increasing their odds of abstaining from cigarette smoking. In addition, readiness to quit measures may appropriately illustrate the effort toward making a behavior change; future studies and smoking cessation programs should consider measuring readiness to quit at several time points to better understand whether readiness to quit fluctuates over time and what resources could aid in successful smoking cessation attempts.
